# Supplementary figures and images for: Systematic genetic mapping of necroptosis identifies SLC39A7 as modulator of death receptor trafficking
Source: Cell Death Differ. 2018 Sep 20;26(6):1138–55. doi: 10.1038/s41418-018-0192-6 (PMC6748104; doi:10.1038/s41418-018-0192-6)

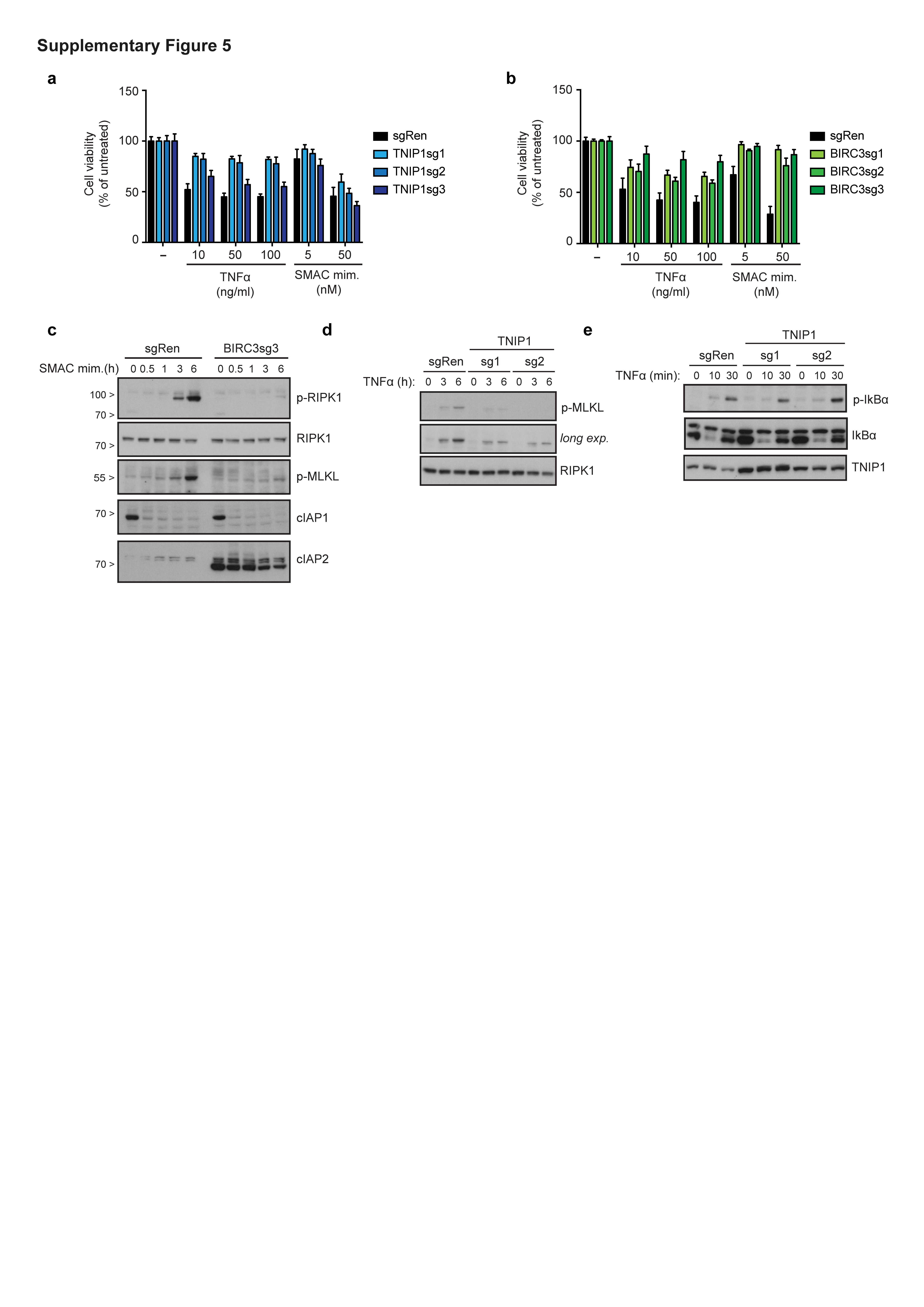

Supplement: Supplementary file 5 — Supplementary Figure 5 [file 41418_2018_192_MOESM5_ESM.tif]
