# Supplementary material for: Systematic genetic mapping of necroptosis identifies SLC39A7 as modulator of death receptor trafficking
Source: Cell Death Differ. 2018 Sep 20;26(6):1138–55. doi: 10.1038/s41418-018-0192-6 (PMC6748104; doi:10.1038/s41418-018-0192-6)
Supplement: Supplementary file 13 — Supplementary figure legends [file 41418_2018_192_MOESM13_ESM.docx]

**CDD-18-0362R: Fauster, Rebsamen et al.**

**SUPPLEMENTARY FIGURE LEGENDS and SUPPLEMENTARY TABLE LEGENDS**

**Supplementary Figure 1: Generation of a *FADD*-deficient KBM7 cell line as novel necroptosis model.** (**a**) KBM7 cells were treated with 10 ng/ml TNFα (T), 0.5 µM SMAC mimetic (S) and 20 µM z-VAD-FMK (Z) for the indicated time. Nec-1s (50 µM) treatment serves as control. Cells were lysed subsequently and subjected to immunoblotting with the indicated antibodies, * indicates non-specific band. (**b**) Human genomic *FADD* locus. The CRISPR target site and sequence as well as localization of the PCR product used to monitor editing efficiency by the T7 Endonuclease I assay are indicated. (**c**) Schematic overview for creation and selection of a KBM7 *FADD^-^* cell line. (**d**) T7 Endonuclease assay to determine *FADD* targeting efficiency. KBM7 cells were harvested at the time points indicated in **c**, and subjected to genomic DNA isolation. The *FADD* locus containing the targeting site was PCR-amplified and the products digested with T7 Endonuclease, followed by agarose gel separation of reaction products. Arrowheads indicate nuclease cleavage products. (**e**) Immunoblot of KBM7 sg*FADD* single cell clones, KBM7 *wildtype* and pooled *FADD­*-targeted cell population serve as reference. (**f**) sg*FADD* single cell clones were treated for the time indicated with 10 ng/ml TNFα. Cells were then lysed and subjected to immunoblotting with the indicated antibodies, * indicates non-specific band; KBM7 *FADD^-^* clone selected for screens is marked in orange in panels **e-f**. (**g**) KBM7 *wildtype* and KBM7 *FADD*^-^ cells were treated for either 3 or 15h with the indicated stimuli. Cells were then lysed and subjected to immunoblotting with the indicated antibodies, ^#^ denotes high number of dead cells/debris in well. (**h**) Schematic overview summarizing the genetic screens presented in this study.

**Supplementary Figure 2: Gene-trap insertion profile and validation of haploid genetic screen hits.** (**a**) Genomic location of gene-trap insertions identified in the combined 100 ng/ml TNFα + 1 µM SMAC mimetic screen affecting the known necroptosis mediators *TNFRSF1A*, *MLKL*, *RIPK1*, and *RIPK3*. Sense integrations (red) generate a gene knockout independent of exonic or intronic position. Antisense integration (blue) targeting exons are disruptive, whereas intronic antisense integration has no effect on gene expression in most cases. (**b**) Cell viability in KBM7 *FADD^-^* lentivirally infected with sgRNAs targeting *RIPK3*, *MLKL*, *TNFR1*, or *Renilla luciferase* (sg*Ren*) as control were treated for 24h with the indicated stimuli. Cell viability was assessed using a luminescence-based readout for ATP (CellTiter Glo). Data represent mean value ± s.d. of two independent experiments performed in triplicates. (**c**) Genomic location of gene-trap insertions (red, sense integration; blue, antisense integration) identified in the indicated haploid genetic screens affecting *SLC39A7*. (**d**) Experimental validation strategy for hits identified in haploid genetic screening approach by CRISPR/*Cas9*-based multicolor competition assay (MCA). KBM7 *FADD^-^* *SpCas9* cells were lentivirally infected with sgRNA-expression LentiGuide-PuroR vectors carrying either an mCherry or GFP fluorescent marker, enabling to monitor the respective targeted cell populations using flow cytometry. sg*Ren-*mCherry control cells were mixed with either sg*Ren*-GFP control or gene targeting sgRNA-GFP cells at 1:1 ratio and subjected to selective pressure with the respective indicated stimuli for 14 days. The percentage of GFP^+^ and mCherry^+^ cells in the remaining viable population was then determined by flow cytometry as a measure for survival/growth differences upon selective pressure. (**e**) MCA of KBM7 *FADD^-^ SpCas9* cells transduced with a GFP marker (GFP^+^) and sgRNAs targeting either *SP3, SPI1, PCBP2*, *LAMTOR1*, or *Renilla luciferase* (*sgRen*) as control, against cells transduced with sg*Ren* and an mCherry marker (mCherry^+^). The cell populations were mixed at 1:1 ratio, treated with SMAC mimetic (1 µM), and analyzed after 14 days by flow cytometry. Data represent mean value ± s.d. of two independent experiments performed in duplicates.

**Supplementary Figure 3: Loss of SLC39A7 leads to an ER stress response.** (**a**) Immunoblot of KBM7 *FADD^-^* *SpCas9* sg*SLC39A7*_618-derived single cell clone. (**b**) HeLa cells were transduced with an inducible lentiviral vector construct harboring C-terminally V5-tagged *SLC39A7*. Cells were induced with 2 µg/ml doxycycline for 24h and analyzed by immunoblotting using the indicated antibodies. (**c**) Confocal microscopy images of doxycycline-induced HeLa cells expressing SLC39A7-V5 immunostained with anti-V5 and Calreticulin (CALR) antibodies. Nuclei were stained with DAPI. Representative cells are shown; scale bars, 10 µm. (**d**) Workflow for membrane-focused proteomics approach. KBM7 *FADD^-^ SLC39A7^-^* or sg*Ren* cells were subjected to Triton X-114 phase separation. Proteins recovered in the detergent phase were precipitated using a methanol/chloroform mixture, resuspended in SDS buffer by sonication and prepared for mass spectrometric analysis by filter-aided sample preparation (FASP). (**e**) Immunoblot of Triton X-114 phase-separated KBM7 *FADD^-^ SLC39A7^-^* or sg*Ren* control samples. Cells were treated as outlined in (**d**) and replicate samples from aqueous and detergent phase immunoblotted using the indicated antibodies, * indicates non-specific band. (**f**) Volcano plot depicting proteins identified by mass spectrometry in KBM7 *FADD^-^ SpCas9 SLC39A7^-^* cells compared to sg*Ren* control. Log2 fold change in abundance of spectral counts (x-axis) is plotted against enrichment significance (y-axis), bubble size corresponds to absolute difference in spectral counts. Proteins with a *p*-value <10^-3^ are labeled by name; proteins with a log2 fold change in spectral counts >1 belonging to GO term “*response to endoplasmic reticulum stress”* are highlighted in red. Data shown are based on two independent experiments, each analyzed as technical duplicates. (**g**) GO terms enriched (adj. *p*-value <0.01) in upregulated proteins (log2fold change >1) in KBM7 *FADD^-^ SpCas9 SLC39A7^-^* cells compared to sg*Ren* control. (**h**) Top hit of GSEA using Hallmark gene sets on significantly up- or downregulated proteins (*p*-value <0.05) in KBM7 *FADD^-^ SpCas9 SLC39A7^-^* cells compared to sg*Ren* control.

**Supplementary Figure 4:** **SLC39A7-deficiency differentially affects death receptor trafficking and signaling.** (**a**) Schematic overview for CRISPR/*Cas9* library screens. The respective target cell lines were infected with an SLC-specific lentiviral CRISPR/*Cas9* library containing 2345 gRNAs targeting 388 SLC genes (~6 guides/gene) as well as 240 control gRNAs. Library-transduced cell populations were either left untreated or exposed to different cell death inducers. After a short period to allow for recovery and outgrowth, cells were harvested, genomic DNA isolated, and sgRNAs enriched by the respective selective pressure were identified via next-generation sequencing by comparison to respective untreated control samples. (**b**) Cell viability of KMB7 *wildtype* and KBM7 *FADD*^-^ cells treated for 24h with TRAIL and 25 µM z-VAD-FMK as indicated. Data represent mean value ± s.d. of two independent experiments performed in triplicates. (**c**) Circos plot of haploid genetic screens in KBM7 cells with 250 ng/ml FASL (red) or 100 ng/ml TRAIL (blue). Each dot represents a mutagenized gene identified in the resistant cell population, the dot size corresponds to the number of independent insertions identified for each gene and the distance from center indicates the significance of enrichment compared to an unselected control data set. Hits with a *p*-value <10^-10^ are labelled by name. (**d**) MCA of KBM7 *SpCas9* cells transduced with a GFP marker (GFP^+^) and sgRNAs targeting either *SLC39A7* or *Renilla luciferase* (sg*Ren*) as control, against cells transduced with sg*Ren* and an mCherry marker (mCherry^+^). The cell populations were mixed in a 1:1 ratio, treated with TRAIL (100 ng/ml), FasL (20 ng/ml), or a combination of TNFα (10 ng/ml) and SMAC mimetic (0.5 µM), and analyzed after 14 days by flow cytometry. Data represent mean value ± s.d. of two independent experiments performed in duplicates, n.d. (not determined) indicates wells with no outgrowth. (**e**) Flow cytometry analysis for FAS surface expression in KBM7 *FADD^-^ SLC39A7^-^* cells reconstituted with either SLC39A7 or GFP. KBM7 *FADD^-^* sg*Ren* and empty KBM7 *FADD^-^ SLC39A7^-^* cells serve as positive and negative control, respectively. Data shown are representative of two independent experiments. (**f**) KBM7 *FADD^-^ SLC39A7^-^* or KBM7 *FADD^-^* cell lysates were incubated for 1h at 37°C in presence or absence of PNGaseF or EndoH, respectively, and analysed by immunoblot with the indicated antibodies. Immunoblots shown are representative of two independent experiments (**g**) Flow cytometry analysis for surface expression of the indicated markers in KBM7 *FADD^-^ SLC39A7^-^* cells and KBM7 *FADD^-^* sg*Ren* control. Data shown are representative of two independent experiments.

**Supplementary Figure 5: sgRNA–mediated transcriptional activation of TNIP1 or BIRC3 inhibits necroptosis.** (**a-b**) Cell viability in KBM7 *FADD^-^* SAM cells transduced with sgRNAs targeting Renilla control, TNIP1 (**a**) or BIRC3 (**b**). Cells were treated as indicated for 24h and viability was assessed using a luminescence-based readout for ATP (CellTiter Glo). Data represent mean value ± s.d. of two (**b**) or three (**a**) independent experiments performed in triplicates. (**c-e**) KBM7 *FADD^-^* cells stably expressing sgRNAs targeting Renilla, BIRC3 (**c**) or TNIP1(**d-e**) were stimulated for the time indicated with Birinapant (0.5 uM in **c**) or TNFα (100ng/ml in **d**; 10 ng/ml in **e**). Cells were then lysed and subjected to immunoblotting with the indicated antibodies. Data shown are representative of two independent experiments.

**Supplementary Table 1: Haploid genetic screen results.** Tables listing the number of identified disruptive insertions per gene, inactivating insertions identified in other genes, total insertions in the control population, *p*-value, and adjusted *p*-value of enrichment for each screen.

**Supplementary Table 2: Proteomics data.** Tables listing the proteins identified by mass spectrometry in KBM7 *FADD^-^ SpCas9 SLC39A7^-^* cells compared to sg*Ren* control, GO term enrichment analysis results in upregulated proteins (log2fold change >1) in KBM7 *FADD^-^ SpCas9 SLC39A7^-^* cells compared to sg*Ren* control, and hits of GSEA using Hallmark gene sets on significantly up- or downregulated proteins (*p*-value <0.05) in KBM7 *FADD^-^ SpCas9 SLC39A7^-^* cells compared to sg*Ren* control.

**Supplementary Table 3: sgRNA sequences used in this study.** Tables listing target genes and the respective sgRNA sequences used.

**Supplementary Table 4: SLC-focused KO genetic screen results.** Table listing the results of the enrichment analysis at sgRNA (DESeq2) and gene (GSEA) level for each screen.

**Supplementary Table 5: sgRNA counts of SLC-focused KO genetic screens.** Table listing sgRNA counts for each screen.

**Supplementary Table 6: Gain-of-function genetic screen results.** Table listing the results of the enrichment analysis at sgRNA (DESeq2) and gene (GSEA) level for each screen.

**Supplementary Table 7: sgRNA counts of the gain-of-function genetic screen.** Table listing sgRNA counts for each screen.
